# Investigation of visual and physical factors associated with inadequate instillation of eyedrops among patients with glaucoma

**DOI:** 10.1371/journal.pone.0251699

**Published:** 2021-05-14

**Authors:** Kenji Kashiwagi, Yuji Matsuda, Yuka Ito, Hisami Kawate, Masako Sakamoto, Shinji Obi, Hirotaka Haro

**Affiliations:** 1 Department of Ophthalmology, Faculty of Medicine, University of Yamanashi, Yamanashi, Japan; 2 Division of Rehabilitation, University of Yamanashi Hospital, Yamanashi, Japan; 3 Department of Nursing, University of Yamanashi Hospital, Yamanashi, Japan; National Taiwan University Hospital, TAIWAN

## Abstract

**Purpose:**

The aim of this study was to investigate the frequency of eyedrop instillation failure and its related physical and visual function factors among glaucoma patients who used hypotensive eyedrops daily.

**Subjects and methods:**

Patients with a history of self-instillation of one or more ocular hypotensive ophthalmic solutions for six or more months were enrolled. Definitions of instillation failure were eyedrop instillation other than on the eye surface; eyedrop contact with eyelashes; eyedrop bottle tip contact with the eyelashes, eye surface or ocular adnexa; or two or more drops instilled with one instillation trial. To clarify factors related to instillation failure, we used visual function tests and investigated cervical spine extension angles during instillation, pinching strength, physical ataxia (evaluated using the Scale for the Assessment and Rating of Ataxia), motor dysfunction of the upper limbs (evaluated using the Disabilities of the Arm, Shoulder and Hand questionnaire), and vision quality (evaluated using the National Eye Institute Visual Function Questionnaire 25).

**Results:**

Of 103 total subjects, 61.2% satisfied the definition of instillation failure. Instillation of the eyedrop other than at the cul-desac (76.2%) was the most frequent reason for failure, followed by contact of the tip of the eyedrop bottle (22.2%) and instillation of the same or more than two eyedrops in a single attempt (11.1%). Advanced age, a shallow cervical spine extension angle, weak pinching strength, poor motor dysfunction of the upper limbs, the degree of ataxia, poor best-corrected visual acuity, and visual field scores were significant risk factors for instillation failure. Vision quality may have some relation to instillation failure.

**Conclusion:**

It is highly recommended that instillation failure be routinely investigated even among patients with adequate experience using eyedrops and that correct therapies are chosen in a patient-based fashion.

## Introduction

Glaucoma is one of the leading ocular diseases resulting in severe visual function loss. Since aging is an important risk factor for glaucoma, it is predicted that the number of glaucoma patients will increase with a progressively aging society [1]. Reduction in intraocular pressure (IOP) is the most effective therapy for glaucoma, and medical treatment with hypotensive ophthalmic solutions is a major therapy for glaucoma. The number of hypotensive ophthalmic solutions increased steadily after the initiation of glaucoma medication [1]. Under these circumstances, a recent study revealed that antiglaucoma medical treatment poses problems, including poor adherence, persistence, and adverse effects [2–6].

Eyedrops cannot exert sufficient effectiveness unless they are properly instilled to the cul-desac, and the incidence of side effects may increase due to improper use, such as instillation around the eyes, instillation of excessive eyedrops, and contamination of the eye-dropper bottle. Proper use of eyedrops is one of the most important points for accurately and safely treating glaucoma.

Various factors have been identified as causes of instillation failure. Davis et al. summarized rates of eyedrop instillation failure as follows: between 18.2 and 80% of patients contaminate their eyedrop bottle by touching their eye or face, 11.3–60.6% instill more than one drop, and 6.8–37.3% miss the eye when attempting to instill a drop [7]. Many factors are reported to be related to instillation failure: older age, insufficient instruction on the eyedrop instillation technique, female sex, arthritis, inadequate posterior bending of the head, much more severe visual-field defects, lack of positive reinforcement to take eyedrops, lower educational level, low self-efficacy, shape and hardness of eyedropper bottles, drug viscosity, and being seen at a clinic rather than a private practice [7–12]. However, previous results were not consistent.

Glaucoma has become prevalent among elderly people who have undergone deterioration of their physical and visual function by aging. Few reports have been thoroughly studied comparing instillation failure of eyedrops with physiological and visual functions. In this study, we investigated the relationship of physical function to instillation failure with the cooperation of orthopedic surgeons and physical therapists (PTs). We also investigated the influence of quality of visual function (QOV) on eyedrop instillation failure using a questionnaire system.

## Patients and methods

This study was performed in accordance with the Helsinki Treaty and approved by the University of Yamanashi Ethical Review Board. All participants gave written informed consent.

### Patients

This study was performed from March 2017 to October 2018. All consecutive adult patients with glaucoma satisfying the inclusion and exclusion criteria were subject to this study. Enrollment criteria were having a history of instillation of one or more ocular hypotensive ophthalmic solutions by themselves for six or more months, best corrected visual acuity of 20/200 or better in the worse eye, and having no difficulty recognizing the tip of an eyedrop bottle when instilling. The exclusion criteria were having ocular complications apparently disturbing eyedrop instillation, such as severe ptosis; having a physical disability, including upper arm, hand, and finger motions; and having a communicative ability that was insufficient to complete the study.

### Criteria for judging instillation failure

Patients were advised to instill one drop of 0.1% hyaluronic acid ophthalmic solution (Hyalein Ophthalmic Solution 0.1%, Santen Pharmaceutical Co., Ltd., Osaka, Japan) to the ocular surface in the same fashion that they usually instilled in the sitting position. Two PTs independently assessed whether patients instilled correctly, and the state of eyedrop instillation was recorded with a camera. According to previous reports [8,11], the definition of instillation failure satisfies any of the following: eyedrop instillation on the ocular adnexa region and not on the cul-desac directly; touching of the tip of the eyedrop bottle to the ocular surface, adnexa tissue, or eyelashes; and instilling two or more eyedrops in a single attempt. If the judgments by two PTs differed, the patients were required to complete additional trials until the judgments of the two PTs were the same. Patients who were assessed to have instillation failure in at least one eye were considered to have unsuccessful cases.

### Ophthalmic examinations

All patients underwent routine ophthalmic examinations within one month before entry, including best corrected visual acuity, slit-lamp examination, and fundus examination. Corrected visual acuity was converted to logMAR (minimum angle of resolution) for the statistical analysis, and the better value for the best corrected visual acuity between the two eyes was employed for the analysis. Visual field testing was performed using the Humphrey Visual Field test program 24–2 (Carl Zeiss Meditec, Inc., Dublin, CA). The latest result of a visual field test performed within three months before entry was employed. Better values of mean deviation (MD) and upper and lower total deviation (TD) between the right and left eyes were subject to statistical analysis.

### Physical examinations

#### Cervical spine extension angle

**Fig 1:**
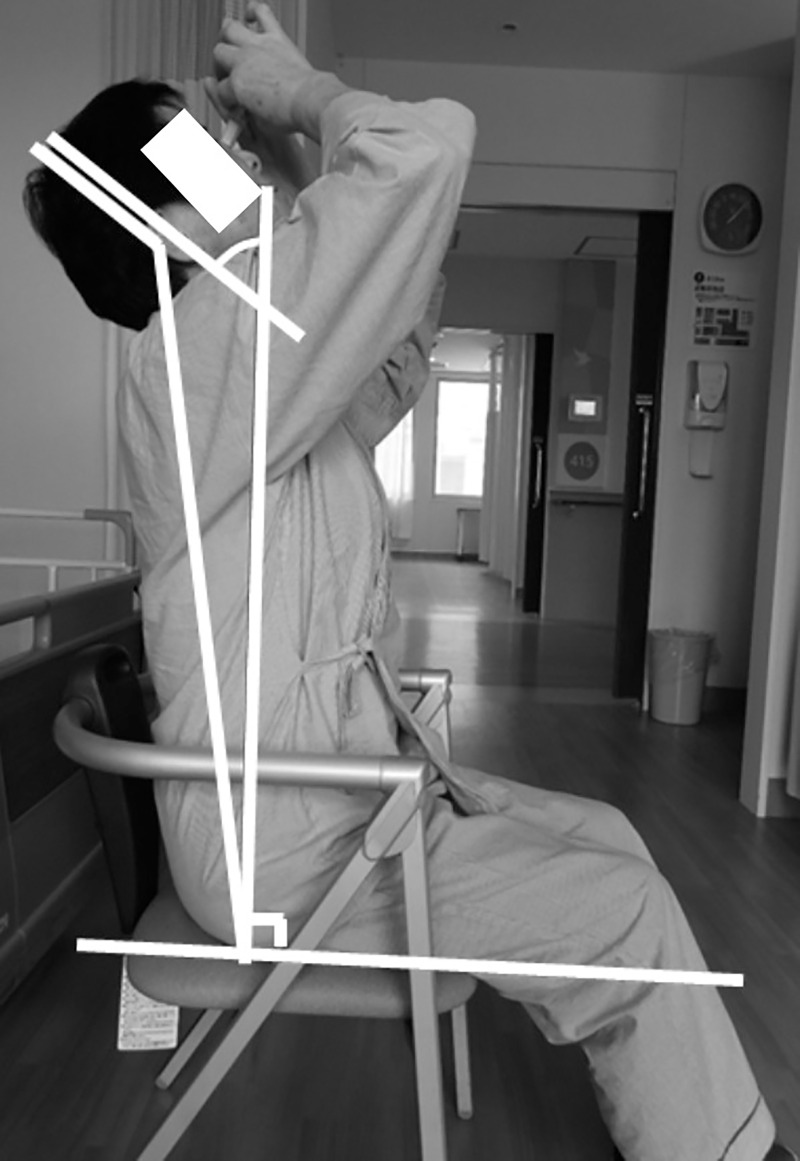
Measurement of the cervical spine extension angle. The cervical spine extension angle (a) is defined as the angle formed by the line connecting the perpendicular to the chair and the parietal auricle.

The position and posture taken by the patient when requiring instillation were photographed from the sagittal plane with a camera, and the angle formed by the line connecting the perpendicular to the chair and the parietal-auricle was determined as the cervical spine extension angle (Fig 1). A participant gave informed consent to be used in Fig 1. A single evaluator measured the cervical spine extension angle using the image analysis program Form Finder^®^ (Form Finder Lab, Inc., Ltd., Tokyo, Japan). This system automatically measures the cervical spine extension angle once the examiner identifies the positions of the neck and buttocks. Next, patients were required to bend their head backward as much as possible in the sitting position to measure the maximum angle of cervical spine extension, and this angle was defined as the maximum cervical spine extension angle. To minimize intermeasurer errors, all subjects were measured in the present study by two examiners who were familiar with the examination. For this reason, we believe that the intermeasurement error is small.

#### Measurement of pinching strength

The pinching strength of the hand on the side used for the eyedrop instillation test was measured with a hydraulic pinch gauge system (Baleline LiTE^®^, Fabrication Enterprises, Inc., Ltd., NY)

#### Examination of ataxia

The status of ataxia was evaluated based on the Scale for the Assessment and Rating of Ataxia (SARA) scaling system, which is widely accepted as a semiquantitative evaluation method for ataxia [13]. This score is an 8-item performance scale including gait, stance, sitting, speech disturbance, finger chase, nose-finger test, fast alternating hand movements, and heel-shin slide, yielding a total score ranging from 0 (no disorder) to 40 (the most severe disorder).

#### Examination of disturbance in upper extremities

The Disabilities of the Arm, Shoulder and Hand (DASH) scaling system was used to evaluate functional disturbances in the upper extremities (http://www.dash.iwh.on.ca/). The DASH scale is designed to evaluate disturbances and measure disabilities of the upper extremities. The main part of the DASH questionnaire is a 30-item disability/symptom scale concerning the patient’s health status during the past week. The scale score ranges from 0 with no disability to 100 with the most severe disability [14].

#### Examination of quality of vision

Vision-specific quality of life (QOV) was assessed with the National Eye Institute Visual Function Questionnaire 25 (NEI-VFQ-25) [15,16]. We used the Japanese version of the NEI-VFQ 25 [17] In brief, the questionnaire has 25 questions for 12 vision-associated aspects of life: general health, general vision, ocular pain, near vision, distant vision, social function, role limitation, dependency, driving, color vision, peripheral vision, and mental health. The subscale scores ranged from 0 to 100, and higher scores indicated better QOL with fewer impairments.

#### Statistical analysis

We compared the investigated factors between the instillation success group and the instillation failure group. A comparative study was conducted using the Mann-Whitney U-test for continuous variables, Spearman’s sign-rank test for rank variables, and the chi-square test or Fisher’s test for categorical variables. Logistic analysis was employed to investigate factors associated with instillation failure. Since numerous factors were investigated, those with continuous variables were divided into two groups by a value showing the highest discrimination ability by univariate analysis. Then, multiple logistic analysis was performed on factors related to instillation failure based on the above two groups. P values less than 0.05 were considered significant. The results are presented as the mean ± standard deviation.

## Results

The total number of enrolled patients was 103, and detailed information is shown in Table 1. The mean age of all subjects was 69.2 ± 8.7 years, and there were 55 male and 48 female patients. The numbers of right hands and left hands used for eyedrop instillation were 96 and 7, respectively.

**Table 1:** Demographics of the enrolled patients.

Parameters	values
# of subjects (M vs F)	103
Age (years)	69.2±8.7
% of male	53.4(55)
Pinching strength (kg)	3.19±1.82
Max cervical spine extension angle (degree)	67.5±14.4
cervical spine extension angle (degree)	52.1±10.8
Better corrected logMAR	0.175±0.372
Better MD (dB)	-11.2±8.4
Better lower TD (dB)	-7.3±6.5
Better upper TD (dB)	-8.5±8.3
SARA scale	2.63±2.79
DASH score	14.3±14.4

M: Male, F: Female, VA: Corrected visual acuity, MAR: Minimum angle of resolution, MD: Mean deviation, TD: Total deviation, SARA: Scale for the Assessment and Rating of Ataxia, DASH: Disabilities of the Arm, Shoulder and Hand scaling system.

### Detailed information on instillation failure

Sixty-three patients (61.2%) were categorized as the instillation failure group, and 27 (26.2%) patients were categorized as having eyedrop instillation failure in both eyes. The rate of instillation failure among right-handed users was 61.4% (59/96), while that of left-handed users was 57.1% (4/7). There was no significant difference in the instillation failure rate between these two groups of users. When analyzing instillation failure, the right eye and the left eye separately, the rates of instillation failure for the right eye and the left eye were 46.6% (48/103) and 43.7% (45/103), respectively. No significant differences in the investigated parameters of instillation failure were observed between the right and left eyes.

### Reasons for instillation failure

Failure to instill eyedrops into the cul-desac was the most frequent (54 patients, 76.2%), with the most frequent cases involving instillation to the lower eyelid (50.5%), followed by the nasal side eyelid (16.7%) and upper eyelid (9.0%). Contact of the tip of the eyedrop bottle with the ocular surface or ocular adnexa region occurred in 14 patients (22.2%), and the majority of the patients (78.6%) touched the eyelashes with the bottle. Seven patients (11.1%) dropped two or more eyedrops, and the numbers of eyedrops at one attempt were two in six patients and three in one patient. All seven cases met any of the other definitions of instillation failure.

### Comparison of the investigated parameters between the instillation success group and failure group

Table 2 shows the results of comparing the investigated parameters between the instillation success group and the failure group. Age was significantly different between the two groups. There was no significant difference in patient sex. The best corrected visual acuity and visual field parameters tended to be worse in the failure group than in the success group. Comparing the relationship between upper and lower TD values and instillation failure, 42.4% of patients with poorer lower TD values failed eye drop instillation, while 51.9% of patients with poorer upper TD values did. These differences were not significant. Among the physical parameters, the instillation failure group showed a significantly weaker pinch strength and shallower cervical spine extension angle at instillation than the success group. The maximum cervical spine extension angles in the instillation failure group showed a tendency to be shallower than those in the success group.

**Table 2:** Comparison of demographics and vision-related parameters between the instillation failure group and the success group.

Parameters	Success	Failure	P value
Age (years)	66.4±8.9	70.9±8.3	0.014*
% of male	52.5	54	0.884
Better corrected logMAR	0.150±0.374	0.191±0.373	0.586
Better MD (dB)	-9.6±8.6	-12.2±8.2	0.127
Better lower TD (dB)	-6.7±5.9	-7.7±7.0	0.53
Better upper TD (dB)	-7.4±7.6	-9.3±8.8	0.427
Pinching strength (kg)	3.73±2.11	2.86±1.53	0.023*
cervical spine extension angle (degree)	55.2±8.1	50.1±11.9	0.024*
Max cervical spine extension angle (degree)	69.4±11.7	66.4±15.9	0.3
SARA scale	1.85±2.25	3.12±3.00	0.018*
DASH score	10.5±10.5	16.8±15.2	0.040*

MAR: Minimum angle of resolution, MD: Mean deviation, TD: Total deviation, SARA: Scale for the Assessment and Rating of Ataxia, DASH: Disabilities of the Arm, Shoulder and Hand scaling system * indicates P<0.05. The Mann-Whitney U-test for continuous variables and Spearman’s sign-rank test for rank variables were applied.

### Correlation between instillation failure and ataxia

The installation failure group showed a worse status of ataxia than the success group, although the SARA scores were relatively low in both groups. The total SARA score in the success group was 1.85 ± 2.25, while that in the instillation failure group was 3.12 ± 3.00, which was significantly different (P = 0.018). In the analysis focused on the motor function of the upper extremities (finger chase, nose-finger test, fast alternating hand movements), the SARA score in the instillation failure group was 1.44 ± 1.64 and that in the success group was 0.87 ± 1.39 (P = 0.064). The instillation failure group showed worse scores on all 8 items except for speech disturbance than did the instillation success group (S1 Table).

### Correlation between instillation failure and disturbance in upper extremities

DASH examination also showed that the instillation failure group had a significantly disturbed ability in the upper extremities compared with that in the success group. The DASH score in the success group was 10.5±10.5, while that in the instillation failure group was 16.8±15.2 (P = 0.040).

### Correlation between NEI-VFQ 25 and instillation failure (Fig 2)

The installation failure group had a worse QOV status than the success group. In all 25 items of the NEI-VFQ investigated, the score was lower in the instillation failure group than in the success group, and the difference between the two groups was significant for all items except general health and ocular pain.

**Fig 2:**
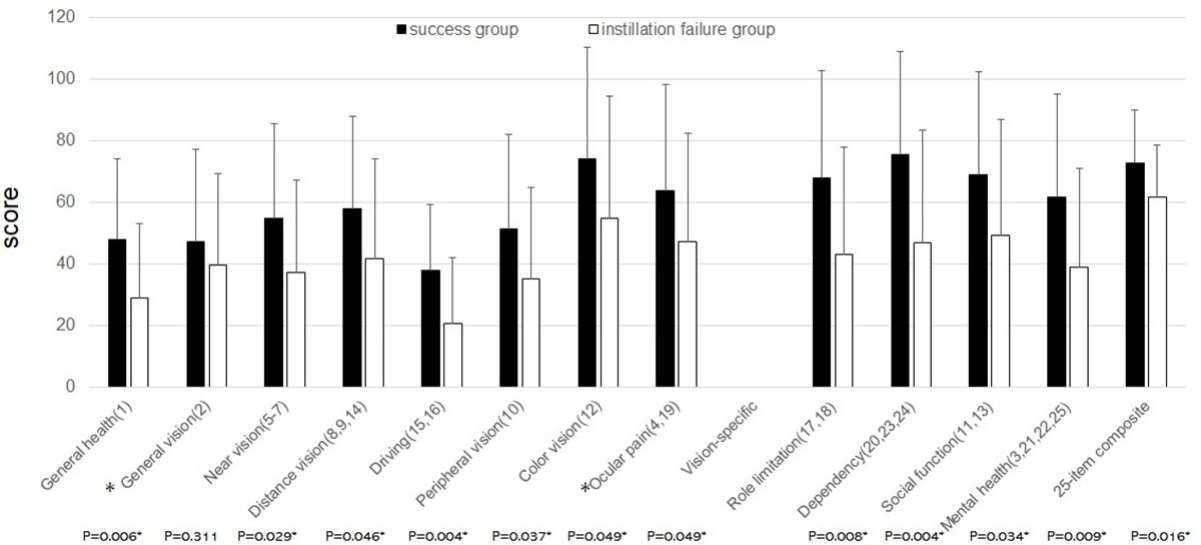
Correlation between NEI-VFQ25 score and rate of instillation failure. Patients belonging to the success group showed significantly higher scores than those in the failure group except for items with* (Mann-Whitney T-test). The number in parentheses represents the item number. NEI-VFQ 25: National Eye Institute Visual Function Questionnaire 25.

### Multiple logistic analysis related to instillation failure (Table 3)

Parameters showing significant associations with instillation failure were total SARA score, upper-extremity SARA score, log MAR, MD, better upper TD, and DASH score. Disturbances in physical function and ataxia as well as visual function worsened instillation failure. S2 Table shows the univariate logistic analysis result. As a subgroup analysis, factors associated with instillation failure were examined in 54 patients whose eyedrops were not placed in the cull-desac. In the multivariate analysis, the SARA total, upper extremities SARA score, logMAR, and better upper TD were extracted as significant parameters, but only the DASH score was not significant.

**Table 3:** Results of the multivariate logistic regression analysis.

Parameter	Discrimination value	Odd ratio	P value
Total SARA score	3	6.43179635	0.001
Upper extremities SARA score	1	13.63944546	0.0002
logMAR	0	15.73201848	< .0001
MD (dB)	-1.4 dB	20.48633844	< .0001
Better upper TD (dB)	-1.7dB	5.799465126	0.011
DASH score	16	8.225459873	0.0034

SARA: Scale for the Assessment and Rating of Ataxia, MAR: Minimum angle of resolution, MD: Mean deviation, TD: Total deviation, DASH: Disabilities of the Arm, Shoulder and Hand.

### Relations among the NEI-VFQ-25, DASH, and SARA examinations

**Fig 3:**
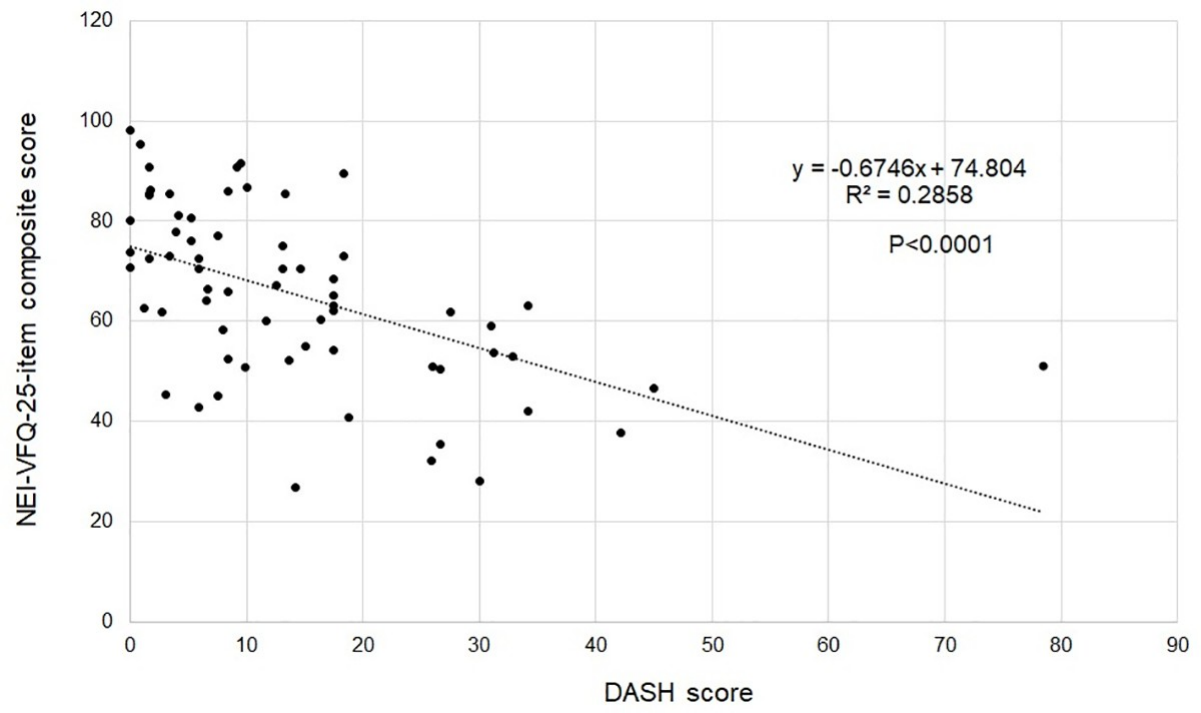
Correlation between NEI-VFQ 25 and DASH examinations. NEI-VFQ 25: National Eye Institute Visual Function Questionnaire 25, DASH: Disabilities of the Arm, Shoulder and Hand.

The NEI-VFQ 25-item composite score showed a significant relationship with the DASH scores, which indicates that QOV function may be deeply related to physical condition status (Fig 3). Other combinations showed no significant relations.

## Discussion

This study revealed that 61.2% of glaucoma patients who used ocular hypotensive eyedrops on a daily basis for more than half a year incorrectly instilled currently challenged ophthalmic solution in at least one eye. Although the instillation failure rate in this study was in the range of those previously reported, it was relatively high. The reason for the high rate of eyedrop instillation failure in this study may be attributed to the fact that the definition of eyedrop instillation failure included the possibility of decreased eyedrop efficacy as well as increased risks of bottle contamination and side effects from the eyedrops. We defined patients instilling two or more eyedrops in a single attempt as those with instillation failure. Multiple eyedrops in a single attempt may increase the incidence of eyedrop-induced adverse effects, but there would be no significant effect on the efficacy of the eyedrops. From this point of view, it may be better to exclude this definition from those of instillation failure in this study, although all seven patients who instilling two or more eyedrops met any of the other definitions of instillation failure.

The most frequent reasons for instillation failure were instillation to an incorrect place and contact of the tip of the eyedropper bottle with the eyelashes and/or eyelids, followed by instillation of two or more drops. Both physical and visual function-related factors were chosen as significant risk factors for instillation failure, such as cervical spine extension angle, finger-pinching strength, ataxia, QOV, visual acuity, and visual field loss.

The rate of instillation failure in previous reports has a relatively wide range (30% to 80%). Although the rate of instillation failure in the current study is similar to that of previous reports, contact of the eyedropper bottle with the eyelid and eyelashes and instillation of multiple drops in a single attempt were much more prevalent than in the current study [7,9,10,18,19].

Previous studies have reported risk factors for instillation failure, such as physical and visual functions, instruction regarding instillation, proficiency in instillation methods, and the properties of eyedropper bottles [7,9,11,19,20].

Many previous papers have reported aging as a risk factor for instillation failure [7,9,11].

The current study also showed aging as a risk factor by univariate analysis. However, in the multiple logistic analysis, aging was not selected as a significant risk factor. Aging deteriorates both physiological and visual function. Instillation failure may be induced not by aging but by physiological or visual functions deeply related to aging. The current study enabled the detection of some physical conditions (such as ataxia) that are significantly related to instillation failure as well as visual function.

The cervical spine extension angle at instillation was significantly correlated with instillation failure in the sitting position. Insufficient head bending may reduce the horizontally projected cross-sectional area of the cornea, which may be a reason for instillation failure. Since many glaucoma patients use prostaglandin (PG) analogs as hypotensive eyedrops and PG often elongates eyelash extension action, it is considered that patients tend to instill eyedrops to the lower target area to avoid contact with elongated eyelashes. These findings may support the fact that many patients instilled eyedrops to the lower eyelid. Since there was no difference in the maximal cervical spine extension angle between the instillation success group and the instillation failure group, it is possible to improve instillation failure by instructing patients to bend their head as much as possible. However, some patients may have difficulty bending their head much more than usual during eye drop instillation because of the decline in their physical performance due to aging. Another option is to adopt a supine position during instillation, as in Naito’s report [8].

Although we excluded patients who had disorders in physical function possibly resulting in instillation failure, pinch strength affected instillation failure. In addition to aging [21], some musculoskeletal or neurological diseases may increase instillation failure. It has been reported that the force required to expel eyedrops sometimes exceeds the ability of some healthy subjects [22] and that the squeezing force of eyedropper bottles is very wide [23,24]. Approximately 20% of ophthalmic patients reported difficulty squeezing their eyedrop bottle. Attention must be paid regarding pinch strength to avoid instillation failure.

We excluded patients with obvious disturbances in this study. The range of DASH among the currently enrolled patients was 0 to 48, which means that the disturbance of the upper extremities was not severe. Despite this enrollment limitation, DASH examination showed that instillation failure patients had significantly poor or impaired upper limb function, which is consistent with previous reports [8,11].

Few reports have investigated the relationship between visual and physical function. We employed the SARA examination, which is widely used to evaluate ataxia. Multiple logistic analysis showed that the total SARA score significantly influenced instillation failure, although the SARA scores in both groups were very low. In the detailed analysis, the instillation failure groups showed worse scores on all 8 items than did the instillation success group, except for speech disturbance.

In the case of subjects with obvious ataxia, it is highly possible that they may have difficulty instilling eyedrops correctly. Since this study aimed to examine whether ataxia affects eye drop instillation failure even in subjects who do not have clear ataxia in practice, we eliminated subjects who had a physical disability including upper arm, hand, and finger motion from the present study. Therefore, the SARA scores of the subjects in this study were mild in terms of the degree of disability and could not significantly interfere with daily life. To the best of our knowledge, this is the first report to reveal that even a degree of ataxia that is not clinically apparent can affect eye drop instillation failure.

It is controversial whether visual function is significantly related to instillation failure. Slean et al. and Tahtham et al. reported no significant correlation [19,20]. In contrast, Naito et al. reported lower corrected visual acuity and inferior hemifield visual field damage were related to instillation failure [11]. The current study showed that some factors related to visual function, including lower best-corrected VA, worse MD, and impairment of upper hemisphere TD, were significantly related to instillation failure. Because the cervical spine extension angle during eye drop instillation is less than 90 degrees, the patient has to look upward to see the tip of the eye drop bottle. However, if the upper visual field is impaired, the patient may not recognize the tip of the eye drop bottle clearly, potentially leading to eye drop instillation failure. There was no significant relation between eye drop instillation failure and MD value or upper or lower TD value in the worse eye. Risk factors for instillation failure reported by Naito et al. are similar to ours except that different hemisphere damage showed a significant relation to instillation failure Naito et al. focused on the more damaged hemisphere, while this study investigated the absolute values of hemisphere TD, which may be influenced by the different methods of comparison. Moreover, there may be differences in the sample background, the degree of visual impairment, and the evaluation method between the two studies. There is no conclusive evidence that the hemisphere affects instillation failure of eyedrops. Further investigations are required to clarify this point.

One of the main aims of glaucoma treatment is to maintain QOV throughout life. Although some items were significantly different between the instillation success and instillation failure groups, there was no significant difference in the 25-item composite between the groups. There may be some correlation between QOV and instillation failure. In the detailed analysis, the instillation failure group showed a significantly lower score for all items on the NEI-VFQ 25 examination, except for items on general vision 2 and ocular pain (Fig 2). Thompson et al. reported that QOV was associated adherence and visual acuity [25]. A reduction in visual function may deteriorate QOV and instillation failure. Alternatively, instillation failure may deteriorate visual function and adverse effects that may be related to QOV.

Few reports have investigated the relationship between visual and physical function and ataxia. The current study showed a significant correlation between the NEI-VFQ-25 and DASH. It is necessary to consider that there is a moderate correlation between quality of vision and physical ability.

The main treatment for glaucoma is medical therapy. It is very important to reduce the chance of instillation failure. Some previous studies have suggested possible methods for improving instillation failure. Lazcano-Gomes et al. recommended proper instruction regarding eyedrop instillation and education to deepen the understanding of medical therapy [26]. Naito et al. suggested adopting a supine instead of sitting position at the time of instillation [8]. The current study indicates some points for improving instillation failure. It is important to instruct patients to take a position with deeper cervical spine extension angles. Improved eyedrop bottles are an important option, such as eyedrop bottles requiring less pinching strength and limiting the dispensed amount to one drop at one attempt. Furthermore, the use of an eye drop instillation aid may improve instillation failure [7,27].

However, there will be some patients who are not able to respond to the abovementioned methods, and the number of patients who have severe disturbances of physical and mental conditions in addition to visual function loss will be increased in an aged society. It should be considered that proper adaptation of surgical approaches and development of new drug-delivery systems requires less patient effort.

There are some limitations to this study. The number of enrolled subjects was not large. We did not examine the instillation failure rate using various types of eyedropper bottles, which may have influenced the results. For this reason, one of the entry criteria was a history of self-instillation of ophthalmic solutions for at least six months. It may be better to prepare age-matched healthy subjects as controls, but it is difficult to prepare such a control group as the patients in this study had used eye drops for a long period of time.

Reduced stereoacuity may contribute to the successful instillation of eyedrops, although we did not investigate stereoacuity in this study. This would be a further task to clarify risk factors for instillation failure of eyedrops. In the present study, we did not compare patients with instillation failure of eye drops in only one eye with those with instillation failure for both eyes due to the small number of patients in each group. One purpose of this study was to determine the rate of eyedrop instillation failure among glaucoma patients who were judged to be familiar with installing ophthalmic solutions. It may be necessary comparing eyedrop instillation failure directly between glaucoma patients and normal subjects under these criteria.

This investigation was a single center trial. Therefore, the current results may not be simply applied to the general population. Therefore, we need to plan further studies to overcome these limitations.

Because the present study was conducted on patients who use glaucoma eye drops on a daily basis, the results cannot simply be applied to all glaucoma patients who use eye drops. However, we believe that long-term glaucoma eye drop users are well accustomed to the act of eye drop instillation. In the present study, the fact that inappropriate ophthalmic instillation was common even among such patients suggests that ophthalmic instillation is not sufficiently practiced among all patients with glaucoma. This indicates the need for new treatment methods, such as a much more effective drug delivery system or other drug administration routes, including oral medicine or injection into the eye, or the development of more safe and effective surgical methods.

There are a variety of risk factors for instillation failure. In addition to the decline in physical ability investigated in this study, various factors, such as cognitive decline, are expected to be associated with age-related instillation failure. Instillation failure results in not only reducing the efficacy of eyedrops but also increasing the chance of adverse effects and economic burden. It is highly recommended to investigate instillation failure and to choose correct therapies in a patient-based fashion.

## Supporting information

S1 Table Comparison of SARA scores by each item between the instillation failure group and the success group. SARA: Scale for the Assessment and Rating of Ataxia, *P = 0.03, Mann-Whitney U-test.
(DOCX)

S2 Table Results of the univariate logistic regression analysis. SARA: Scale for the Assessment and Rating of Ataxia, MAR: Minimum angle of resolution, MD: Mean deviation, TD: Total deviation, DASH: Disabilities of the Arm, Shoulder and Hand, NEI-VFQ 25: National Eye Institute Visual Function Questionnaire 25.
(DOCX)

## References

[1] Sakamoto M, Kitamura K, Kashiwagi K. Changes in Glaucoma Medication during the Past Eight Years and Future Directions in Japan Based on an Insurance Medical Claim Database. J Ophthalmol 2017; 2017: 7642049. 10.1155/2017/7642049 29225966 PMC5684567

[2] Kashiwagi K, Furuya T. Persistence with topical glaucoma therapy among newly diagnosed Japanese patients. Jpn J Ophthalmol 2014; 58: 68–74. 10.1007/s10384-013-0284-2 24408788

[3] Tsumura T, Kashiwagi K, Suzuki Y, Yoshikawa K, Suzumura H, et al. A nationwide survey of factors influencing adherence to ocular hypotensive eyedrops in Japan. Int Ophthalmol 2018. 10.1007/s10792-018-0820-7 29330811

[4] Friedman DS, Okeke CO, Jampel HD, Ying GS, Plyler RJ, et al. Risk factors for poor adherence to eyedrops in electronically monitored patients with glaucoma. Ophthalmology 2009; 116: 1097–1105. 10.1016/j.ophtha.2009.01.021 19376591

[5] Nordstrom BL, Friedman DS, Mozaffari E, Quigley HA, Walker AM. Persistence and adherence with topical glaucoma therapy. Am J Ophthalmol 2005; 140: 598–606. 10.1016/j.ajo.2005.04.051 16226511

[6] Okeke CO, Quigley HA, Jampel HD, Ying GS, Plyler RJ, et al. Adherence with topical glaucoma medication monitored electronically the Travatan Dosing Aid study. Ophthalmology 2009; 116: 191–199. 10.1016/j.ophtha.2008.09.004 19084273

[7] Davis SA, Sleath B, Carpenter DM, Blalock SJ, Muir KW, et al. Drop instillation and glaucoma. Curr Opin Ophthalmol 2018; 29: 171–177. 10.1097/ICU.0000000000000451 29140818 PMC6422028

[8] Naito T, Yoshikawa K, Namiguchi K, Mizoue S, Shiraishi A, et al. Comparison of success rates in eye drop instillation between sitting position and supine position. PLoS One 2018; 13: e0204363. 10.1371/journal.pone.0204363 30235323 PMC6147506

[9] Hennessy AL, Katz J, Covert D, Protzko C, Robin AL. Videotaped evaluation of eyedrop instillation in glaucoma patients with visual impairment or moderate to severe visual field loss. Ophthalmology 2010; 117: 2345–2352. 10.1016/j.ophtha.2010.03.040 20580092

[10] Stone JL, Robin AL, Novack GD, Covert DW, Cagle GD. An objective evaluation of eyedrop instillation in patients with glaucoma. Arch Ophthalmol 2009; 127: 732–736. 10.1001/archophthalmol.2009.96 19506189

[11] Naito T, Namiguchi K, Yoshikawa K, Miyamoto K, Mizoue S, et al. Factors affecting eye drop instillation in glaucoma patients with visual field defect. PLoS One 2017; 12: e0185874. 10.1371/journal.pone.0185874 29023521 PMC5638255

[12] Yoshikawa K, Yamada H. Influence of container structures and content solutions on dispensing time of ophthalmic solutions. Clin Ophthalmol 2010; 4: 481–486. 10.2147/opth.s10804 20535225 PMC2879350

[13] Schmitz-Hubsch T, du Montcel ST, Baliko L, Berciano J, Boesch S, et al. Scale for the assessment and rating of ataxia: development of a new clinical scale. Neurology 2006; 66: 1717–1720. 10.1212/01.wnl.0000219042.60538.92 16769946

[14] Hudak PL, Amadio PC, Bombardier C. Development of an upper extremity outcome measure: the DASH (disabilities of the arm, shoulder and hand) [corrected]. The Upper Extremity Collaborative Group (UECG). Am J Ind Med 1996; 29: 602–608. 10.1002/(SICI)1097-0274(199606)29:6&lt;602::AID-AJIM4&gt;3.0.CO;2-L 8773720

[15] Mangione CM, Lee PP, Pitts J, Gutierrez P, Berry S, et al. Psychometric properties of the National Eye Institute Visual Function Questionnaire (NEI-VFQ). NEI-VFQ Field Test Investigators. Arch Ophthalmol 1998; 116: 1496–1504. 10.1001/archopht.116.11.1496 9823352

[16] Mangione CM, Lee PP, Gutierrez PR, Spritzer K, Berry S, et al. Development of the 25-item National Eye Institute Visual Function Questionnaire. Arch Ophthalmol 2001; 119: 1050–1058. 10.1001/archopht.119.7.1050 11448327

[17] Suzukamo Y, Oshika T, Yuzawa M, Tokuda Y, Tomidokoro A, et al. Psychometric properties of the 25-item National Eye Institute Visual Function Questionnaire (NEI VFQ-25), Japanese version. Health and Quality of Life Outcomes 2005; 3: 65. 10.1186/1477-7525-3-65 16248900 PMC1283746

[18] Gupta R, Patil B, Shah BM, Bali SJ, Mishra SK, et al. Evaluating eye drop instillation technique in glaucoma patients. J Glaucoma 2012; 21: 189–192. 10.1097/IJG.0b013e31820bd2e1 21336146

[19] Sleath B, Blalock S, Covert D, Stone JL, Skinner AC, et al. The relationship between glaucoma medication adherence, eye drop technique, and visual field defect severity. Ophthalmology 2011; 118: 2398–2402. 10.1016/j.ophtha.2011.05.013 21856009 PMC3223548

[20] Tatham AJ, Sarodia U, Gatrad F, Awan A. Eye drop instillation technique in patients with glaucoma. Eye (Lond) 2013; 27: 1293–1298. 10.1038/eye.2013.187 23970024 PMC3831141

[21] Ranganathan VK, Siemionow V, Sahgal V, Yue GH. Effects of aging on hand function. J Am Geriatr Soc 2001; 49: 1478–1484. 10.1046/j.1532-5415.2001.4911240.x 11890586

[22] Winfield AJ, Jessiman D, Williams A, Esakowitz L. A study of the causes of noncompliance by patients prescribed eyedrops. Br J Ophthalmol 1990; 74: 477–480. 10.1136/bjo.74.8.477 2390523 PMC1042177

[23] Kashiwagi K. Wide Variation of Squeezing Force and Dispensing Time Interval among Eyedropper Bottles. J Ophthalmol 2019; 2019: 7250563. 10.1155/2019/7250563 31143473 PMC6501172

[24] Connor AJ, Severn PS. Force requirements in topical medicine use—the squeezability factor. Eye (Lond) 2011; 25: 466–469. 10.1038/eye.2011.5 21293499 PMC3171228

[25] Thompson AC, Woolson S, Olsen MK, Danus S, Bosworth HB, et al. Relationship between electronically measured medication adherence and vision-related quality of life in a cohort of patients with open-angle glaucoma. BMJ Open Ophthalmol 2018; 3: e000114. 10.1136/bmjophth-2017-000114 29657978 PMC5895971

[26] Lazcano-Gomez G, Castillejos A, Kahook M, Jimenez-Roman J, Gonzalez-Salinas R. Videographic Assessment of Glaucoma Drop Instillation. J Curr Glaucoma Pract 2015; 9: 47–50. 10.5005/jp-journals-10008-1183 26997834 PMC4750026

[27] Davies I, Williams AM, Muir KW. Aids for eye drop administration. Surv Ophthalmol 2017; 62: 332–345. 10.1016/j.survophthal.2016.12.009 28011246

