# Supplementary material for: Investigation of visual and physical factors associated with inadequate instillation of eyedrops among patients with glaucoma
Source: PLoS One. 2021 May 14;16(5):e0251699. doi: 10.1371/journal.pone.0251699 (PMC8121298; doi:10.1371/journal.pone.0251699)
Supplement: S1 Table — SARA: Scale for the Assessment and Rating of Ataxia, *P = 0.03, Mann-Whitney U-test. (DOCX) [file pone.0251699.s001.docx]

| Supplemental table 1 | |  |  |  |  |  |  |  |  |  |  |  |
| --- | --- | --- | --- | --- | --- | --- | --- | --- | --- | --- | --- | --- |
| Item | Gait | Stance | Sitting | Speech disturbance | Finger chase | Finger chase | Nose-finger test | Nose-finger test | Fast alternating hand movement (left hand) | Fast alternating hand movement (right hand) | Heel-shin slide | Heel-shin slide |
|  |  |  |  |  | (left hand) | (right hand) | (left hand) | (right hand) |  |  | (left leg) * | (right leg) |
| Success | 0.95±1.49 | 0.31±0.66 | 0.00±0 | 0.00±0 | 0.13±0.34 | 0.10±0.31 | 0.23±0.43 | 0.08±0.27 | 0.00±0 | 0.00±0 | 0.03±0.16 | 0.03±0.16 |
| Failure | 1.50±1.76 | 0.48±0.85 | 0.02±0.13 | 0.00±0 | 0.23±0.43 | 0.20±0.41 | 0.28±0.45 | 0.17±0.38 | 0.03±0.25 | 0.03±0.25 | 0.13±0.34 | 0.03±0.18 |
| p-value | 0.066 | 0.201 | 0.321 | N.A.! | 0.138 | 0.134 | 0.493 | 0.123 | 0.321 | 0.323 | 0.039^*^ | 0.83 |
