# Supplementary material for: Investigation of visual and physical factors associated with inadequate instillation of eyedrops among patients with glaucoma
Source: PLoS One. 2021 May 14;16(5):e0251699. doi: 10.1371/journal.pone.0251699 (PMC8121298; doi:10.1371/journal.pone.0251699)
Supplement: S2 Table — SARA: Scale for the Assessment and Rating of Ataxia, MAR: Minimum angle of resolution, MD: Mean deviation, TD: Total deviation, DASH: Disabilities of the Arm, Shoulder and Hand, NEI-VFQ 25: National Eye Institute Visual Function Questionnaire 25. (DOCX) [file pone.0251699.s002.docx]

| Supplemental Table 2 |  |  |  |  |
| --- | --- | --- | --- | --- |
|  | **Odd ratio** | **Lower 95% of CI** | **Upper 95% CI** | **P Value** |
| Total SARA score | 1.21 | 1.03 | 1.45 | 0.018 |
| (point) |  |  |  |  |
| Upper extremities SARA score (point) | 1.29 | 0.98 | 1.74 | 0.065 |
| age(year) | 1.06 | 1.04 | 1.12 | 0.014 |
| Pinching strength | 0.761 | 0.59 | 0.95 | 0.023 |
| (kg) |  |  |  |  |
| cervical spine extension angle (degree) | 0.95 | 0.91 | 0.99 | 0.024 |
| logMAR | 1.36 | 0.46 | 4.39 | 0.586 |
| (unit) |  |  |  |  |
| MD (dB) | 0.96 | 0.91 | 1.01 | 0.128 |
| Better lower TD | 0.99 | 0.94 | 1.06 | 0.941 |
| (dB) |  |  |  |  |
| Better upper TD | 0.99 | 0.94 | 1.04 | 0.822 |
| (dB) |  |  |  |  |
| DASH | 1.06 | 0.98 | 1.19 | 0.153 |
| (point) |  |  |  |  |
| VFQ25 | 0.99 | 0.94 | 1.03 | 0.632 |
| (point) |  |  |  |  |
| Sex | 1.06 | 0.48 | 2.35 | 0.884 |
| (M/F) |  |  |  |  |
